# Identity-related experiences of Asian American trainees in gynecologic oncology

**DOI:** 10.1016/j.gore.2022.101097

**Published:** 2022-11-06

**Authors:** Jhalak Dholakia, Yeon Woo Lee, Karen H. Lu, Warner K. Huh, S. Diane Yamada, Katherine C. Fuh, Amanika S. Kumar, Margaret I. Liang, Navya Nair, Kenneth H. Kim

**Affiliations:** a Division of Gynecologic Oncology, Department of Obstetrics and Gynecology, University of Alabama at Birmingham, Birmingham, AL, United States; b Department of Obstetrics and Gynecology, Boston University Medical Center, Boston, MA, United States; c Department of Gynecologic Oncology and Reproductive Medicine, The University of Texas MD Anderson Cancer Center, Houston, TX, United States; d Division of Gynecologic Oncology, Department of Obstetrics and Gynecology, University of Chicago, Chicago, IL, United States; e Division of Gynecologic Oncology, Department of Obstetrics and Gynecology, University of California, San Francisco, San Francisco, CA, United States; f Division of Gynecologic Oncology, Department of Obstetrics and Gynecology, Mayo Clinic, Rochester, MN, United States; g Division of Gynecologic Oncology, Department of Obstetrics and Gynecology, Cedars-Sinai Medical Center, West Hollywood, CA, United States; h Division of Gynecologic Oncology, Department of Obstetrics and Gynecology, Louisiana State University, New Orleans, LA, United States

## Abstract

**Background:**

Anti-Asian violence increased during the COVID-19 pandemic. Asian American/Pacific Islanders (AAPI) represent a diverse population experiencing a long history of stereotyping and exclusionism; however, this group is often left out of diversity/inclusion conversations. In academic medicine, AAPI are under-represented in leadership. We characterized the personal/professional experiences of AAPI gynecologic oncology trainees and assessed the impact of a virtual panel discussion with leaders in the field.

**Methods:**

An anonymous survey was disseminated online to trainees in/interested in gynecologic oncology fellowship who identified as AAPI, using modified snowball sampling. A virtual session with AAPI leaders in gynecologic oncology discussed themes emerging from survey responses. Session attendees completed an anonymous follow-up survey. Results were assessed quantitatively and qualitatively.

**Results:**

44/59 (75%) respondents participated in the pre-survey; 23 (39%) participated in the virtual session. All session participants (23/23, 100%) completed the post-session survey. Participants reported increased identity-related thoughts with the COVID-19 pandemic (88% during, 61% prior). Sixty-eight percent reported that identity-related thoughts/awareness changed during the pandemic. Presence of AAPI colleagues was associated with higher perceived identity-related support from their department. Of those without AAPI coworkers, none (0%) felt ‘moderately’ or ‘extremely well supported.’ Qualitative analysis demonstrated that the panel discussion created a sense of community and encouragement, combating previously reported isolation and self-consciousness. Participants reported more connection with their heritage and identified more personal/professional topics that might be related to their cultural backgrounds.

**Discussion:**

This intervention demonstrates the opportunity to provide a supportive network for mentorship and professional development in a culturally inclusive way.

## Highlights

• Asian Americans (AA) have a complex history of immigration and inclusion to the United States.
• AA GYN oncology and OB/GYN trainees reported safety & isolation concerns re: anti-Asian violence and the COVID-19 pandemic.
• A panel session with AA leaders in SGO increased personal and professional perceptions and interests related to identity.
• A lack of Pacific Islander representation indicates a need to better reach this demographic in gynecologic oncology.
• Mentorship incorporating cultural backgrounds may contribute to efforts in professional diversity & equity.

## 1 Introduction

In 2020, as the COVID-19 pandemic spread, anti-Asian sentiments and violence spread alongside the virus. A steady rise in hate crimes against Asian people came to a head during the Atlanta spa shootings in March 2021 which took the lives of 8 people, 6 of whom were women of Asian-American descent (Jeung, 2021). Since that time, this trend has continued with multiple violent attacks against AAPI (Asian American and Pacific Islander) individuals and another shooting in May 2022 in Dallas. Abuse, fear, and stark racism have also extended into healthcare: one study reported that 46 % of Asian surgical residents experienced discrimination in the workplace stemming from race/ethnicity (Ruiz et al., 2021, Yuce et al., 2020).

The AAPI identity and experience in the United States has historically been fraught with exclusionism and xenophobia. Beginning with the 1875 Page Act and the 1882 Chinese Exclusion Act, anti-Asian laws targeted the immigration and employment of specific ethnic groups. Anti-Asian sentiments continued into the 1940s, when President Roosevelt authorized the internment of over 120,000 Japanese Americans into concentration camps due to perceived national security threats. Native Hawaiian and Pacific Islanders uniquely contend with otherization and erasure in their own ancestral lands as a result of colonialism and are often overlooked due to conflation into the larger AAPI demographic.

Despite this history, AAPI people are often a “forgotten” minority in discussions related to diversity and inclusion in many institutions and political spheres. Instead, the model minority myth, which lumps the extremely heterogeneous and diverse ethnicities and cultures under a singular, monolithic label, has perpetuated harmful stereotypes while being disguised in a compliment. Stereotypically, AAPI people are seen as passive, well-behaved, obedient, and submissive, ultimately lacking leadership characteristics. These biases have clear impacts in the workplace, with AAPI workers being the least likely of all racial groups to be promoted to management and leadership roles across different industries (Gee and Peck, 2018). This pattern persists in academic medicine, where AAPI and particularly Pacific Islanders are less likely to advance to full professor or department chair and are under-represented in leadership compared to the overall proportion of AAPI physicians (Choi and Rustgi, 2021).

In this study, we characterized the personal and professional experiences of AAPI trainees in gynecologic oncology, and we assessed the impact of a virtual panel discussion with AAPI leaders in the field.

## 2 Methods

This project was IRB-approved at Cedars-Sinai Medical Center. An anonymous survey was disseminated online to trainees in or interested in gynecologic oncology fellowship who identified as AAPI, using modified snowball sampling. Modified snowball sampling is often used in social sciences research for smaller or difficult to access populations and enables initially approached participants to recruit additional participants. Trainees who had expressed concerns or desire for AAPI support to leaders in the field were initially recruited and encouraged to include colleagues and contacts who may benefit, Participants who completed the initial (‘pre’) survey were invited to a 90-minute virtual panel discussion with AAPI leaders in gynecologic oncology (‘intervention’). Participants of this session were provided an anonymous follow-up (‘post’) survey. Pre and post responses were not matched; both surveys included quantitative and qualitative components. No incentives were provided. A descriptive analysis was performed for quantitative results. The authors developed an initial coding framework around which to assess qualitative results. Major themes were identified in the codified data and summarized using participant narrative responses. A descriptive analysis was also performed on the type and frequency of words and themes from participant feedback.

In the virtual session held on 11/18/2021, a panel of 8 leaders in gynecologic oncology who identified as AAPI participated in discussion on three themes: leadership and networking, model minority and AAPI identity, and clinical environment and impact. These themes emerged from questions and concerns provided by participants in the pre-survey. Participant concerns and comments were provided to the panel members prior to the discussion. These leaders chose amongst themselves regarding which discussant would lead each theme; however, all panel members were able to contribute to all discussions during the event. The session was planned for one hour duration. All participants were together for the duration (no ‘break-out rooms’) to create a collaborative environment and allow all trainees and leaders to interact with each other.

## 3 Results

### 3.1 Quantitative Responses

Of 59 approached individuals, a total of 44 (75 %) respondents participated in the pre-survey and 23 (39 %) participated in the virtual session. All session participants (23/23, 100 %) completed the post-session survey (Table 1). Most pre-session survey participants identified as *cis*-gendered female (68 %), of Asian American heritage (93 %), and belonging to second generation (natively born to immigrant parents) immigration groups (57 %). Most participants were either gynecologic oncology fellows (53 %) or OB/GYN residents (39 %.) Of note, no participants (0 %) identified as Pacific Islander.

**Table 1:** Pre-Session Survey Demographics.

	**n = 44 (%)**
**Gender Identity**	
Cis Female	30 (68 %)
Cis Male	11 (25 %)
Prefer not to answer	3 (7 %)
**Racial/Ethnic Identity**	
Asian American	41 (93 %)
Two or More Races	2 (5 %)
Other	1 (2 %)
**Immigration Generation**	
First Generation	15 (34 %)
Second Generation	25 (57 %)
Third Generation	2 (5 %)
Fourth Generation or Beyond	2 (5 %)
**Training/Career Position**	
Gynecologic Oncology Fellow	23 (53 %)
OB/GYN Resident	17 (39 %)
Graduate from Fellowship	2 (5 %)
Resident in Another Specialty	1 (2 %)
Fellow in Another Specialty	1 (2 %)

**Fig. 1:**
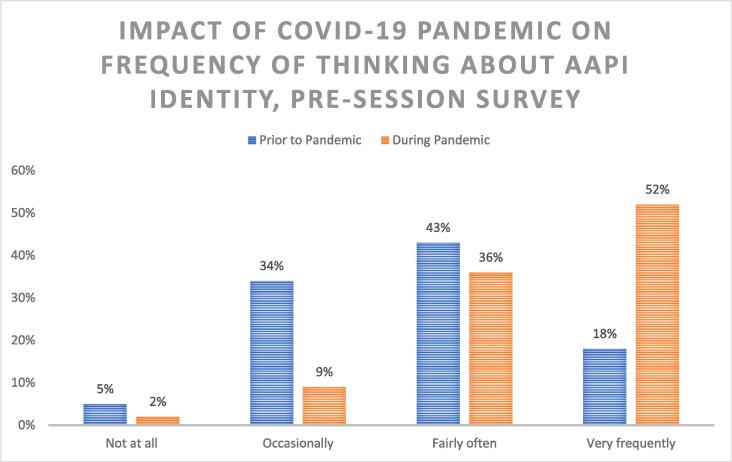
Impact of COVID-19 Pandemic on Frequency of Thinking About AAPI Identity, Pre-Session Survey. The COVID-19 pandemic was associated with increased frequency of thinking about AAPI identity.

**Fig. 2:**
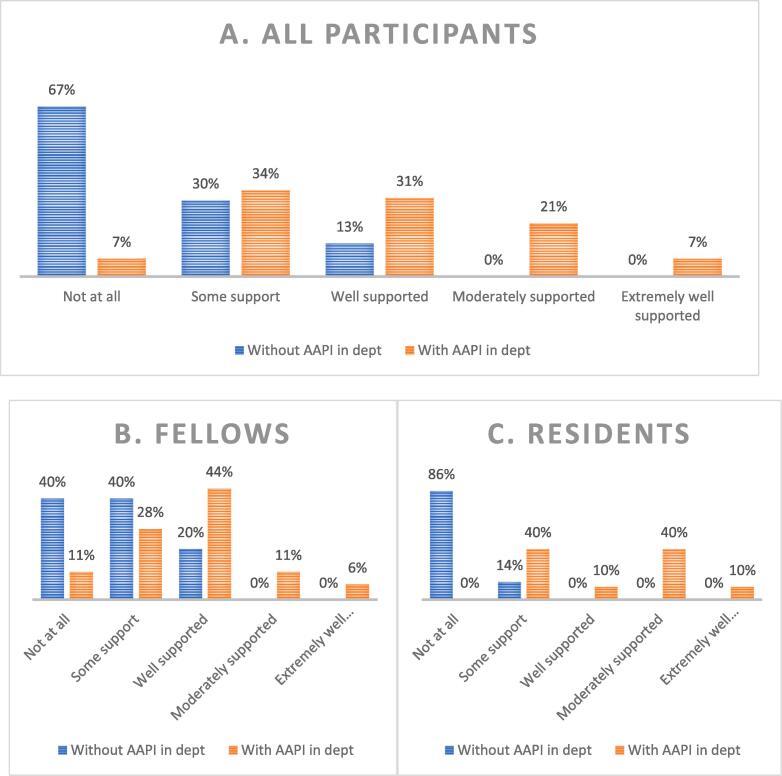
Presence of AAPI Colleagues and Association with Perceived Support Related to Identity, Pre-Session Survey. A) All respondents. B) Fellow respondents. C) Resident respondents. In all groups, presence of other AAPI in the department was associated with increased perceived support related to identity. Of the participants who did not have other AAPI coworkers, none felt ‘moderately’ or ‘extremely well supported.’.

Participants reported increased frequency of thoughts related to AAPI identity associated with the onset of the COVID-19 pandemic: 88 % had these thoughts ‘fairly often’ or ‘very frequently’ during the pandemic compared to 61 % prior to the pandemic (Fig. 1). Sixty-eight percent reported that their thoughts and awareness related to their identity changed over the course of the pandemic. The majority reported that their department had at least one other AAPI provider (66 %). Presence of AAPI colleagues was associated with higher perceived support from their department with respect to participant identity (Fig. 2). Conversely, of the participants who did not have other AAPI coworkers, none (0 %) felt ‘moderately’ or ‘extremely well supported.’.

**Fig. 3:**
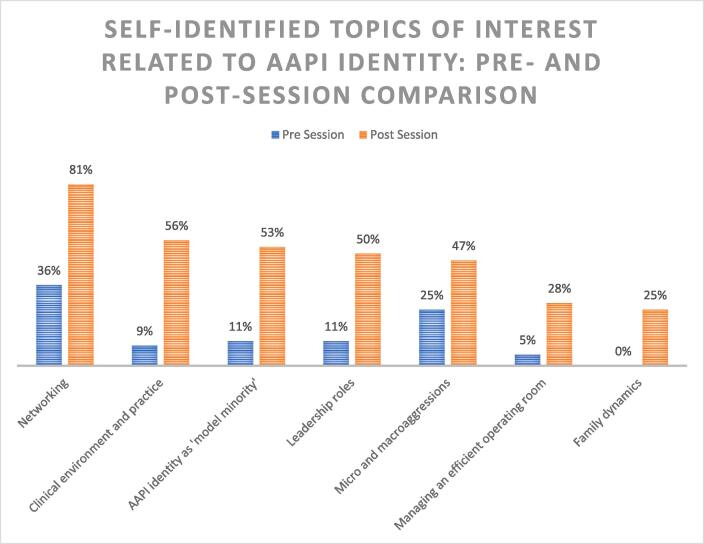
Self-Identified Topics of Interest Related to AAPI Identity: Pre and Post Session Comparison. Participants identified more topics of interest related to AAPI identity in both personal and professional spheres following the panel session.

**Fig. 4:**
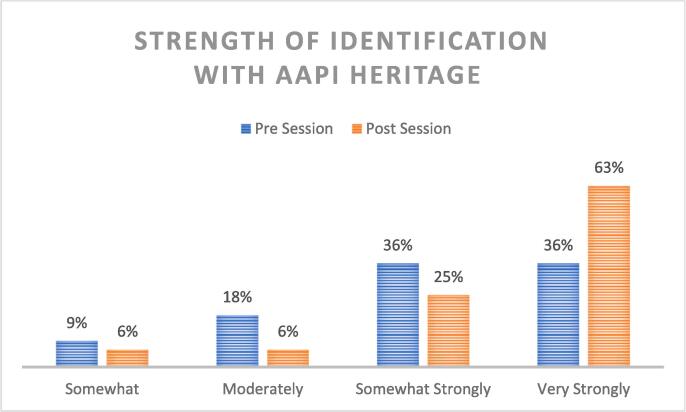
Strength of Identification with AAPI Heritage: Pre and Post Session Survey Comparison. Participants more strongly identified with AAPI heritage following the panel session.

Participants were also surveyed regarding topics of interest that may relate to AAPI identity (Fig. 3.) Respondents demonstrated increased interest in professionally-oriented topics (networking, clinical practice, leadership) as well as personal concepts (‘model minority’, micro/macroaggressions, family dynamics) after the panel discussion. Respondents also identified more strongly with AAPI identity following the discussion (Fig. 4.).

### 3.2 Qualitative Responses

#### 3.2.1 Pre-event responses and themes

**Fig. 5:**
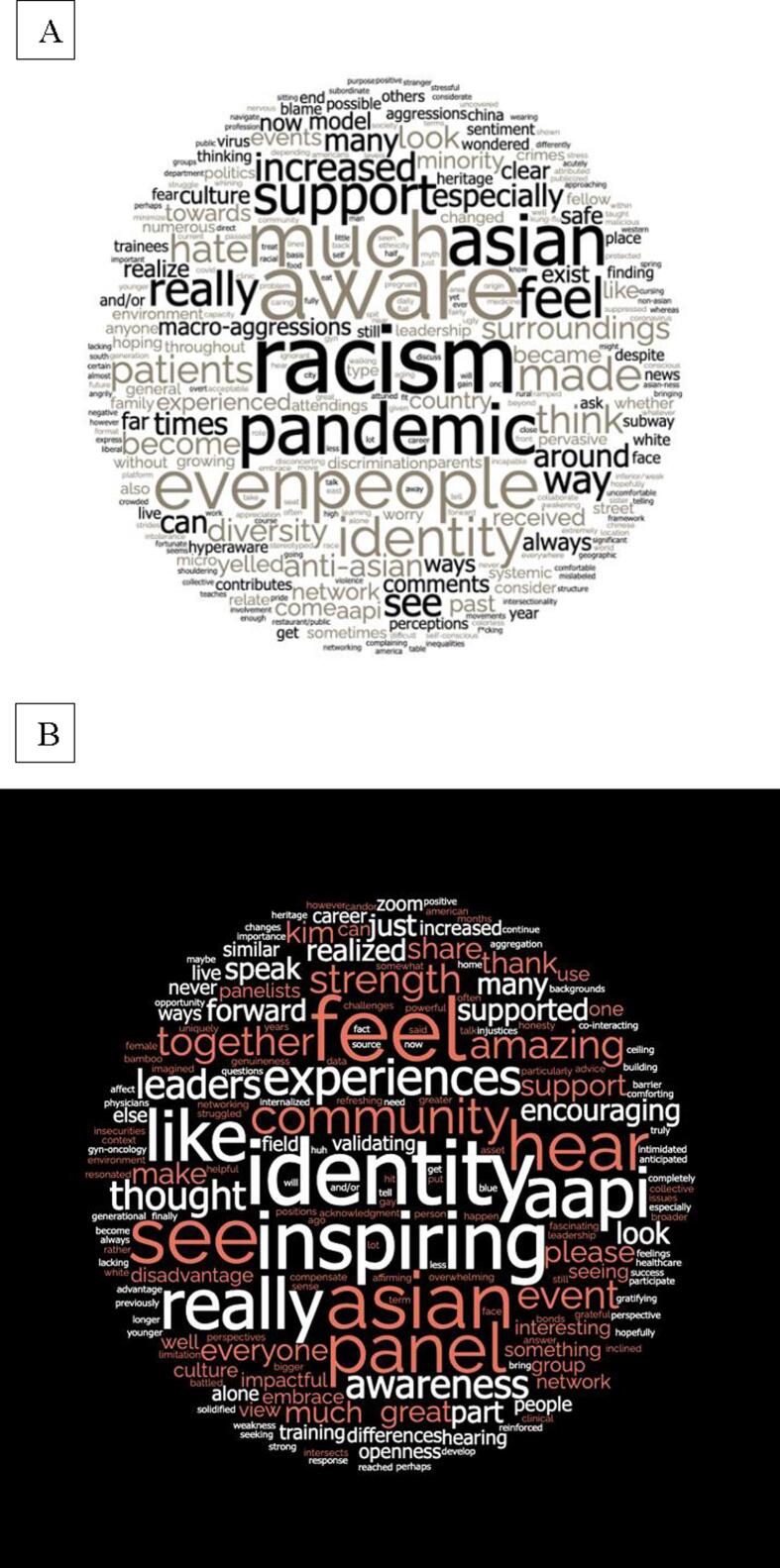
Word Clouds of Qualitative Feedback. (A) Pre-session themes centered on the pandemic, racism, safety concerns, and isolation. (B) Post-session themes centered on community and identity as a source of strength.

Participants were asked about recent changes to their beliefs and awareness regarding AAPI identity and given the option to provide qualitative feedback. Thirty participants provided qualitative responses as part of the pre-session survey and the following themes were noted (Fig. 5A.).

1. Concern for physical safety

A number of participants (n = 8) discussed how the pandemic and the acts of physical and verbal violence towards people of AAPI descent made them feel unsafe or fearful for themselves, their AAPI patients, and their families. One participant noted: “I am hyperaware of my surroundings and don’t always feel safe, depending on where I am*.*” Another provided detailed descriptions of discrete acts of racism related to their AAPI identity, like being verbally harassed or spit at by strangers. Participants expressed feelings of hypervigilance, stress, and anxiety as a direct result with one participant noting, “I was nervous to take the subway… I felt protected while wearing a mask/hat/sunglasses because people wouldn’t see as much of my face.”.

2. Awareness of their AAPI identity and anti-Asian racism

Many participants (n = 14) noted an increase in their overall ‘awareness’ about their identity, stemming from a recognition of the racist acts directed towards people of AAPI descent. One participant shared, “This pandemic was an ‘awakening’ for my AAPI identity.” Another stated, “I am acutely aware of [my heritage] with all the Asian hate and discrimination I have seen. I think about it far more than I ever have.” For some, this manifested in how they thought their patients perceived them as their provider: “I wondered if patients felt a certain way towards me because of the way I looked.” Some participants (n = 3) expressed that macro- and microaggressions directed towards them both in public and in the workplace led to increases in their self-consciousness. It was also shared that experiencing macroaggressions from their own attendings helped them recognize that “there is a systemic racism in this country that has been uncovered by the pandemic.”.

3. Lack of support or community

A few participants (n = 3) expressed feelings of being “alone” with regards to their AAPI identity, describing a lack of a support system or network. One participant shared, “It is difficult to navigate without any other people around me at work that look like me, or that can relate.” Participants expressed a desire to obtain increased support and establish a wider network through the event: “I am hoping this event will lead to…a networking and support framework for us all.”.

#### 3.2.2 Post-session responses and themes

Following the virtual panel session, a survey was administered to all participants to assess how thoughts and awareness about their identity had changed because of the event. Twenty-three participants (100 % of those that participated in the panel) provided qualitative responses and the following themes were noted (Fig. 5B.).

1. Sense of community and togetherness

More than half of participants (n = 13) reported that the event solidified a sense of community and support that they were lacking prior to the event. Many participants shared that they “no longer felt alone” and expressed interest in further strengthening and developing this community. Participants expressed feelings of “comfort”, “affirmation”, and “validation” that stemmed from being part of a larger community. One participant noted: “This event made me feel like I am part of a bigger network of people who have similar backgrounds and experiences as me. I cannot tell you how impactful and inspiring this was.”.

2. AAPI identity as a source of strength

For some participants (n = 6), the event helped them recognize their AAPI identity as a source of strength rather than weakness. Some participants felt that the event served as a trigger to recognize that in the past, they had felt the opposite. One noted: “I think it finally hit home that I saw being ‘Asian’ as a ‘disadvantage,’ and had completely ‘internalized it” and another participant echoed these sentiments: “I always thought of being Asian as a weakness in the US I had to compensate for but now I see it as a strength.” For some, this served as a motivation to “use [their] awareness of [their AAPI] identity as a strength in [their] career and training.”.

3. Perspectives from leaders

Some participants (n = 2) commented on the “candid”, “genuine”, “honest”, and “open” ways that the panelists shared their experiences during the event. One stated, “I realized many of us share similar feelings about the challenges and/or injustices that Asians face. It was very refreshing and encouraging to hear from leaders in our field who have battled with these insecurities as well.” AAPI representation in the gynecologic oncology leadership was meaningful to the participants. One participant noted that it was “amazing to see our [AAPI] leaders embrace our collective identity, answer our questions, and share experiences” and another stated that it is “inspiring to see people that look like [them] in the leadership positions [which] makes [them] realize that [they] could do it, too.” Many participants (n = 8) used words like “inspiring,” “encouraging,” “positive,” and “validating” to describe the event.

## 4 Discussion

Anti-Asian hate and discrimination have increased since 9/11 and the COVID-19 pandemic, contributing to stressors on minority individuals. More than one-third of Asian Americans have changed their daily schedules due to concerns of race-based violence, with concerns that healthcare screening and maintenance are decreasing in this population (Chen, 2022). Efforts to promote diversity and safe work environments, including representative participation in leadership positions, are necessary to promote culturally inclusive healthcare and research that improves patient equity (Cohen et al., 2002). The impact of xenophobia during the COVID-19 pandemic has prompted AAPI individuals and communities to raise their voice in these conversations.

AAPI identity was developed in the 1960 s to facilitate social and political advocacy for its members. However, this group encompasses hundreds of ethnic identities and cultural backgrounds, with various languages, histories, cultures, and religions. Furthermore, these populations were incorporated into the United States via multiple ways including elective and coerced immigration as well as colonization. An individual’s immigration ‘generation’ also impacts their experience. While all are counted as ‘American,’ their inclusion in one large population demographic is reductive. Stereotyping and concepts such as the model minority myth have furthered division both within the community and with other minority groups; these trends internalize self-doubt and have been associated with detrimental mental health for AAPI young adults (Cheng et al., 2017). In the professional sphere, Asians are less likely to be promoted to leadership positions and are less likely to be identified as natural leaders (Gee and Peck, 2018).

In this intervention, we found that COVID-19-related anti-Asian sentiments increased identity awareness and safety concerns for OB/GYN and gynecologic oncology trainees both personally and professionally. The presence of AAPI colleagues was correlated with increased feelings of support, underscoring the importance of diversity in creating an inclusive workplace. A virtual panel discussion by Asian American leaders in gynecologic oncology created a sense of community and encouragement, combating previously reported isolation and self-consciousness. After this session, participants reported more connection with their heritage and identified more personal and professional topics that might be related to their cultural backgrounds. This intervention demonstrates the opportunity to provide a supportive network for mentorship and professional development in a culturally inclusive way.

Limitations include methods of participant recruitment, relatively limited sample size and lack of long-term follow-up at this time. Modified snowball sampling is often used in sociologic research in hard-to-capture populations; however, this necessitates self-identification and risks excluding those who may not be involved in the AAPI community. This method does pose the risk of selection bias: for one potential example, please see the discussion of Pacific Islander participants below. In future efforts, avenues to reach small or under-represented groups include using SGO or similar organization rosters as well as using social media to reach a more comprehensive group of participants. This program was not initially planned as a longitudinal intervention; however, future diversity and mentorship efforts may benefit from a timeline and assessment plan that could capture long-term impact. Furthermore, there was moderate participant attrition throughout the pre-survey and session/post-survey phases: improving participation throughout and identifying causes for attrition may be valuable.

Notably, no respondents or panel leaders identified as “Pacific Islander (PI),” indicating poor penetration into this demographic. Native Hawaiian and Pacific Islanders (NHPI) are under-represented in medicine and have unique health disparities and experiences that merit targeted attempts at inclusion in future studies. Their experiences related to colonialism and cultural erasure, and the impact of these factors on providers and patients alike, should also be recognized. Further mentorship attempts should be more cognizant in reaching and recognizing NHPI as a unique group. Disaggregation of AAPI identities should also be implemented moving forward to identify inter-group disparities and challenges. (Of note in this study, questions were framed as AAPI-inclusive and therefore this nomenclature was used throughout for consistency).

This intervention offers a model to develop mentorship and networking relationships within medical organizations that respects and incorporates identity and cultural factors that are often taboo in the professional world. By building relationships based on common experiences and cultural backgrounds, both positive and negative, trainees and early career faculty can benefit from the lessons learned by more senior members. Incorporating individual experiences and recognizing the mentee’s perspective are key components of effective mentorship (Boitano et al., 2021).

Valuing representation and developing programs to promote equity within our organizations represent a concrete example of commitment to diversity that promotes an inclusive work community. Such representation has been demonstrated to improve rapport and trust in minority and historically under-served communities, thereby facilitating a collaborative environment that can better care for patients. Institutions and professional societies would benefit from networking and mentorship groups that address these needs.

Conflict of Interest.

The authors report no pertinent conflicts of interest for this publication. Dr Huh accepts consulting fees from Roche Diagnostics and AstraZeneca and leadership for Inovio and Parexel and serves as a medicolegal expert. Dr Yamada serves as a leader for the Society of Gynecologic Oncology. Dr Liang reports grants from the NCI (P30CA013148) and NCCIH (U24AT011310). Dr Nair discloses a relationship with the 10.13039/100016163Women’s Foundation.

## Declaration of Competing Interest

The authors declare that they have no known competing financial interests or personal relationships that could have appeared to influence the work reported in this paper.
